# The association of biomarkers of iron status with peripheral arterial disease in US adults

**DOI:** 10.1186/1471-2261-9-34

**Published:** 2009-08-03

**Authors:** Andy Menke, José Manuel Fernández-Real, Paul Muntner, Eliseo Guallar

**Affiliations:** 1 Department of Epidemiology, Johns Hopkins Bloomberg School of Public Health, Baltimore, USA; 2 Department of Diabetes, Endocrinology, and Nutrition, Institut d'Investigació Biomédica de Girona, Girona, Spain; 3 CIBEROBN Fisiopatología de la Obesidad y Nutrición, Girona, Spain; 4 Department of Community and Preventive Medicine, Mount Sinai School of Medicine, New York, USA; 5 Department of Cardiovascular Epidemiology and Population Genetics, National Center for Cardiovascular Research (CNIC), Madrid, Spain

## Abstract

**Background:**

Several studies have examined the association of biomarkers of iron metabolism with measures of carotid artery atherosclerosis, with inconsistent results. Few studies, however, have evaluated the association between biomarkers of iron metabolism and peripheral arterial disease (PAD). The purpose of this study is to examine the association of ferritin and transferrin saturation with PAD.

**Methods:**

Serum ferritin, transferrin saturation, and PAD, defined as having an ankle-brachial blood pressure index <0.9, were measured in 1,631 men and 1,031 postmenopausal women participating in the 1999-2002 National Health and Nutrition Examination Survey (NHANES).

**Results:**

The multivariable adjusted odds ratios (95% confidence interval) for PAD associated with a two-fold increase in serum ferritin and transferrin saturation were 1.18 (1.00-1.41) and 1.45 (0.83-2.51), respectively, for men and 1.04 (0.87-1.25) and 1.55 (0.98-2.45), respectively, for women. After stratifying by cholesterol levels, the multivariable adjusted odds ratios (95% confidence intervals) for PAD associated with a two-fold increase in ferritin and transferrin saturation was 1.04 (0.78-1.39) and 0.73 (0.35-1.50), respectively, for men with total cholesterol <200 mg/dL and 1.30 (0.99-1.72) and 2.59 (0.99-6.78), respectively, for men with total cholesterol ≥ 200 mg/dL (p-value for interaction was 0.58 for ferritin and 0.08 for transferrin saturation). After stratifying by cholesterol levels, the multivariable adjusted odds ratios (95% confidence intervals) for PAD associated with a two-fold increase in ferritin and transferrin saturation was 0.66 (0.41-1.05) and 0.75 (0.44-1.28), respectively, for women with total cholesterol <200 mg/dL, and 1.20 (0.95-1.51) and 2.07 (1.01-4.22), respectively, for women with total cholesterol ≥ 200 mg/dL (p-value for interaction was 0.05 for ferritin and 0.02 for transferrin saturation).

**Conclusion:**

In this large nationally representative sample of men and postmenopausal women, we found a modest association of ferritin and transferrin saturation with PAD, particularly among those with high cholesterol levels.

## Background

Peripheral arterial disease (PAD), a condition characterized by advanced atherosclerosis in the lower extremities [1], affects 5% of US adults aged ≥ 40 years and 18% of US adults aged ≥ 75 years [2]. Individuals with atherosclerosis in the lower extremities are likely to have atherosclerosis in other arteries placing them at an increased risk of coronary heart disease, stroke, and all-cause mortality [3-6]. Iron has been hypothesized to promote atherosclerosis based on its ability to catalyze the generation of highly reactive oxygen species, such as hydroxyl radicals, through Fenton and Haber-Weiss reactions [7]. There is no consensus in the literature regarding the role of iron in the development of atherosclerosis [8]. Several studies have examined the association of biomarkers of iron metabolism with measures of carotid artery atherosclerosis, with inconsistent results [9-13]. Few studies, however, have evaluated the association between biomarkers of iron metabolism and PAD [8].

The purpose of the current analysis was to evaluate the association of serum ferritin and transferrin saturation with the prevalence of PAD, assessed through the ankle-brachial blood pressure index (ABI), using data from the 1999-2002 National Health and Nutrition Examination Survey (NHANES 1999-2002).

## Methods

### Study Population

NHANES 1999-2002 was a stratified, multistage probability survey designed to select a representative sample of the civilian non-institutionalized US population [14]. NHANES 1999-2002 measured ABI in participants aged ≥ 40 years. Of 6,671 adults aged ≥ 40 years who participated in the interview and examination, we excluded 1,475 premenopausal women, 846 participants with anemia (non-Hispanic black men: hemoglobin <12.5 g/dL; other men: hemoglobin <13.5 g/dL; non-Hispanic black women: hemoglobin <11 g/dL; other women: hemoglobin <12 g/dL), 506 participants taking iron supplements, 10 participants with likely hemochromatosis (men: serum iron >190 μg/dL, serum ferritin >300 ng/mL, and transferrin saturation >60%; women: serum iron >175 μg/dL, serum ferritin >200 ng/mL, and transferrin saturation >60%), 123 participants with an ABI ≥ 1.3 (a sign of severe arterial stiffness) [4], 486 participants missing data for ABI, 50 participants missing data for ferritin, 23 participants missing data for transferrin saturation, and 490 participants missing other covariate information, the final sample included 2,662 participants (n = 1631 men and n = 1031 women). Using alternative cutoff points for excluding participants with anemia did not materially alter the results of the study. When participants excluded because of missing data were compared to those included in the study, excluded participants were more likely to be older, non-Hispanic black, and to have a higher body mass index, lower cholesterol levels, and a higher prevalence of diabetes. The protocol for NHANES 1999-2002 was approved by the National Center for Health Statistics of the Centers for Disease Control and Prevention Institutional Review Board. All participants gave written informed consent.

### Data Collection

Data were collected during an in-home interview and a subsequent visit to a mobile examination center. During the in-home interview, information on demographic, household income, education, smoking status, alcohol consumption, and medication use was collected using a standardized questionnaire.

Height and weight were measured and body mass index (BMI) was calculated as weight in kilograms divided by height in meters squared. Systolic blood pressure was determined based on the average of 3 blood pressure measurements. Participants had a blood specimen drawn from their antecubital vein by a trained phlebotomist according to a standardized protocol. Serum C-reactive protein (CRP) was quantified using latex-enhanced nephelometry, a high sensitivity assay. Alanine aminotransferase (ALT) and total serum cholesterol was measured enzymatically. High-density lipoprotein (HDL) cholesterol was measured using a heparin-manganese precipitation method. Serum creatinine was measured by a kinetic rate Jaffe method. We calculated estimated glomerular filtration rate (eGFR) using the Modification of Diet and Renal Disease equation after aligning the serum creatinine concentrations with the assay employed in the development of the equation [15,16]. Individuals were classified as having an eGFR ≥ 90, 60-89, or <60 ml/min/1.73 m^2^. We defined diabetes mellitus as a self-reported history of diabetes with concurrent use of anti-diabetes medication.

### Markers of Iron Metabolism

A detailed description of the measurement of ferritin and transferrin saturation is available elsewhere [14]. Ferritin was measured using a single-incubation two-site immunoradiometric assay (Bio-Rad Laboratories, Hercules, CA). The software used was not able to quantify ferritin levels below 5 ng/mL; therefore, participants with ferritin levels below 5 ng/mL (n = 2) were assigned a value of 3 ng/mL. The inter-assay coefficient of variation ranged from 2.4% to 5.7% in NHANES 1999-2000 and 3.8% to 5.1% in NHANES 2001-2002. Serum iron and total iron-binding capacity were measured using a modification of the automated AAII-25 colorimetric method on an Alpkem Flow Solutions IV system (Alpkem, Inc., College Station, TX). Transferrin saturation was calculated as (iron/total iron-binding capacity)*100%.

### Peripheral Arterial Disease

Systolic blood pressure measurements for ABI determination were taken via blood pressure cuff on the right brachial artery and both posterior tibial arteries with the participant lying supine on the exam table. These measurements were taken separately from the measurements to determine blood pressure. Participants with conditions preventing measurement in the right arm had the left brachial artery systolic blood pressure measured instead (n = 8). ABI measurements were taken twice for participants aged 40-59 years and once for participants aged ≥ 60 years. Right and left ABI were calculated as the ratio of the mean systolic blood pressure in the respective ankle to the mean brachial systolic blood pressure in the arm. Participants with an ABI <0.9 in either leg were considered to have PAD. The 0.9 cutoff value for ABI has 95% sensitivity for detecting angiogram positive disease and almost 100% specificity in excluding healthy individuals [17].

### Statistical Analysis

All analyses were conducted separately for men and postmenopausal women. Participants were categorized into gender-specific tertiles of ferritin and transferrin saturation based on the weighted population distribution. Age and race-ethnicity adjusted means and percentages were calculated by linear regression for continuous covariates and logistic regression for dichotomous covariates.

We calculated the odds ratios of PAD associated with tertile of ferritin and transferrin saturation after multivariable adjustment using logistic regression. Initial models adjusted for age (continuous) and race-ethnicity (non-Hispanic white, non-Hispanic black, Mexican-American, other). Subsequent models further adjusted for household income (<$20,000 and ≥ $20,000), high school education, smoking status (current, former, never), alcohol consumption (<1 and ≥ 1 drink per week), and body mass index (<25, 25-29, and ≥ 30 kg/m^2^). Additional models further adjusted for systolic blood pressure (continuous), high sensitivity C-reactive protein (log-transformed, continuous), ALT (log-transformed, continuous), total cholesterol (continuous), high-density lipoprotein-cholesterol (continuous), estimated glomerular filtration rate (<60, 60-89, and ≥ 90 ml/min/1.73 m^2^), diabetes mellitus, use of blood pressure medication, diabetes medication, aspirin, and hormone replacement therapy (among women). Tests for linear trend across tertiles of ferritin and transferrin saturation were computed by including an ordinal variable with the median of each tertile of ferritin or transferrin in the regression models. Further analyses included ferritin and transferrin saturation as continuous, log-transformed variables. For these analyses, we present odds ratios for PAD associated with a two-fold increase in ferritin or transferrin saturation. The analysis of ferritin and transferrin saturation as continuous, log-transformed variables was repeated after stratifying by total cholesterol levels above and below 200 mg/dL to investigate the potential interaction with cholesterol levels as has been reported in previous studies [11,18-20].

Data were analyzed using SUDAAN (version 9.0; Research Triangle Institute, Research Triangle Park, NC) to account for the complex NHANES sampling design, including unequal probabilities of selection, over-sampling, and non-response.

## Results

The geometric mean ferritin and transferrin saturation levels were 148 ng/mL and 25.8%, respectively, for men and 81 ng/mL and 21.9%, respectively, for women. The prevalence of PAD was 4% for men and 8% for post-menopausal women. Among men, PAD was associated with being older, being non-Hispanic black, having low income, being a current smoker, having higher CRP, having lower ALT, and having an eGFR <60 ml/min/1.73 m^2 ^(Table 1). Among women, PAD was associated with being older, being non-Hispanic black, being a former smoker, having a lower HDL-cholesterol level, and having an eGFR <60 ml/min/1.73 m^2^.

**Table 1:** Age and race-ethnicity adjusted participant characteristics* by peripheral arterial disease status and gender

	Men (n = 1,631)			Women (n = 1,031)		
	PAD n = 109 4%†	No PAD n = 1575 96%†	p-value	PAD n = 110 8%†	No PAD n = 921 92%†	p-value

Age, years	67 (1.4)	54 (0.3)	<0.001	70 (1.4)	62 (0.4)	<0.001
Non-Hispanic white, %	73 (5.1)	78 (1.9)	0.32	73 (4.7)	78 (2.6)	0.19
Non-Hispanic black, %	22 (4.6)	8 (0.8)	<0.001	17 (4.6)	8 (1.4)	0.005
Mexican-American, %	3 (1.3)	5 (0.8)	0.29	3 (1.1)	3 (0.7)	0.94
Low income, %	31 (6.0)	15 (1.2)	0.003	35 (5.1)	26 (2.1)	0.10
High school education, %	74 (4.2)	79 (1.7)	0.14	72 (6.5)	77 (1.8)	0.45
Current smokers, %	56 (6.9)	23 (1.5)	<0.001	33 (5.1)	19 (1.6)	0.09
Former smokers, %	32 (5.6)	39 (1.4)	0.28	35 (5.4)	24 (2.2)	0.05
Consume alcohol, %	43 (9.0)	45 (2.4)	0.79	23 (6.5)	21 (2.0)	0.82
Body mass index ≥ 30 kg/m^2^, %	30 (5.9)	30 (1.4)	0.92	45 (5.9)	34 (2.5)	0.07
C-reactive protein, mg/L	3.6 (2.7-4.8)	1.8 (1.7-2.0)	<0.001	3.5 (2.8-4.5)	2.9 (2.7-3.2)	0.17
Alanine aminotransferase, U/L	23 (22-25)	27 (27-28)	<0.001	19 (18-21)	20 (20-21)	0.39
Systolic blood pressure, mm Hg	130 (3.1)	126 (0.6)	0.21	138 (2.2)	134 (0.8)	0.12
Total cholesterol, mg/dL	222 (9.8)	211 (2.2)	0.24	215 (4.4)	221 (1.3)	0.18
HDL-cholesterol, mg/dL	45 (1.5)	46 (0.5)	0.64	53 (1.7)	59 (0.9)	0.005
eGFR <60 ml/min/1.73 m^2^, %	14 (3.6)	8 (0.7)	0.04	28 (3.6)	16 (1.4)	0.04
Diabetes mellitus, %	11 (2.8)	7 (0.6)	0.13	8 (2.1)	7 (0.9)	0.74
Use of antihypertensive medication, %	29 (3.8)	21 (1.3)	0.05	44 (5.6)	35 (2.3)	0.12
Use of lipid lowering medication, %	17 (3.8)	15 (1.4)	0.65	26 (6.9)	16 (1.3)	0.15
Use of aspirin, %	21 (4.4)	17 (1.6)	0.29	22 (5.2)	16 (1.7)	0.26
Use of HRT, %	-	-	-	21 (5.1)	31 (2.1)	0.10
Ferritin, ng/mL	159 (135-188)	148 (141-157)	0.44	85 (72-101)	81 (76-86)	0.60
Transferrin saturation, mean percent	25 (23-28)	26 (25-27)	0.49	23 (21-25)	22 (21-22)	0.23

HDL: High-density lipoprotein; eGFR: Estimated glomerular filtration rate; HRT: Hormone replacement therapy

*Mean or percentage (standard error) except for C-reactive protein, alanine aminotransferase, ferritin and transferrin saturation which are geometric mean (95% confidence interval)

†Weighted percentage of population

Among men, the multivariable adjusted odds ratios (95% confidence intervals) for PAD comparing the highest to the lowest tertiles of ferritin and transferrin saturation were 1.87 (0.96-3.63) and 1.48 (0.82-2.64), respectively (Table 2). The multivariable adjusted odds ratios (95% confidence intervals) for PAD associated with a two-fold increase in ferritin and transferrin saturation were 1.18 (1.00-1.41) and 1.45 (0.83-2.51), respectively. After stratifying by cholesterol levels, the multivariable adjusted odds ratios (95% confidence intervals) for PAD associated with a two-fold increase in ferritin and transferrin saturation was 1.04 (0.78-1.39) and 0.73 (0.35-1.50), respectively, for men with total cholesterol <200 mg/dL and 1.30 (0.99-1.72) and 2.59 (0.99-6.78), respectively, for men with total cholesterol ≥ 200 mg/dL (p-value for interaction was 0.58 for ferritin and 0.08 for transferrin saturation).

**Table 2:** Adjusted odds ratios (95% confidence intervals) of peripheral arterial disease by tertile of and for a two-fold increase in ferritin and transferrin saturation stratified by gender

	Tertile 1	Tertile 2	Tertile 3	p-trend	Two-fold increase
Men - ferritin	<109 ng/mL (3%)*	109-221 ng/mL (5%)*	≥ 222 ng/mL (4%)*		

Age and race-ethnicity adjusted	1.00	1.70 (1.07 - 2.69)	1.38 (0.69 - 2.77)	0.53	1.07 (0.89 - 1.27)
Multivariable model 1†	1.00	1.76 (1.04 - 2.96)	1.29 (0.63 - 2.62)	0.71	1.05 (0.87 - 1.27)
Multivariable model 2‡	1.00	2.13 (1.07 - 4.24)	1.87 (0.96 - 3.63)	0.16	1.18 (1.00 - 1.41)

Men - transferrin saturation	<22.4% (5%)*	22.4-30.4% (4%)*	≥ 30.5% (4%)*		

Age and race-ethnicity adjusted	1.00	0.90 (0.44 - 1.86)	0.82 (0.45 - 1.47)	0.49	0.87 (0.54 - 1.41)
Multivariable model 1†	1.00	0.82 (0.39 - 1.73)	0.87 (0.52 - 1.46)	0.61	0.94 (0.59 - 1.48)
Multivariable model 2‡	1.00	1.11 (0.54 - 2.29)	1.48 (0.82 - 2.64)	0.19	1.45 (0.83 - 2.51)

Women - ferritin	<57 ng/mL (9%)*	57-115 ng/mL (7%)*	≥ 116 ng/mL (9%)*		

Age and race-ethnicity adjusted	1.00	0.69 (0.40 - 1.18)	0.87 (0.51 - 1.51)	0.81	1.05 (0.88 - 1.26)
Multivariable model 1†	1.00	0.61 (0.34 - 1.09)	0.82 (0.47 - 1.41)	0.70	1.04 (0.86 - 1.26)
Multivariable model 2‡	1.00	0.52 (0.28 - 0.99)	0.77 (0.46 - 1.30)	0.65	1.04 (0.87 - 1.25)

Women - transferrin saturation	<19.0% (9%)*	19.0-26.0% (7%)*	≥ 26.1% (9%)*		

Age and race-ethnicity adjusted	1.00	0.69 (0.40 - 1.21)	1.00 (0.64 - 1.57)	0.84	1.26 (0.81 - 1.95)
Multivariable model 1†	1.00	0.69 (0.41 - 1.17)	1.11 (0.71 - 1.73)	0.55	1.34 (0.85 - 2.12)
Multivariable model 2‡	1.00	0.82 (0.44 - 1.56)	1.29 (0.79 - 2.10)	0.23	1.55 (0.98 - 2.45)

*Weighted percentage of population with peripheral arterial disease

†Adjusted for age (continuous), race-ethnicity (non-Hispanic white, non-Hispanic black, Mexican-American, and other), household income (<$20,000 and ≥ $20,000), high school education, smoking status (current, former, and never), alcohol consumption (<1 and ≥ 1 drink per week), and body mass index (<25, 25-29, and ≥ 30 kg/m^2^)

‡Adjusted for variables in model 1 and systolic blood pressure (continuous), C-reactive protein (log-transformed, continuous), alanine aminotransferase (log-transformed, continuous), total cholesterol (continuously), high-density lipoprotein-cholesterol (continuous), estimated glomerular filtration rate (<60, 60-89, and ≥ 90 ml/min/1.73 m^2^), diabetes mellitus, blood pressure medication, cholesterol medication, aspirin, and hormone replacement therapy (among women)

Among women the multivariable adjusted odds ratios (95% confidence intervals) for PAD comparing the highest to the lowest tertiles of ferritin and transferrin saturation were 0.77 (0.46-1.30) and 1.29 (0.79-2.10), respectively (Table 2). The multivariable adjusted odds ratios (95% confidence intervals) for PAD associated with a two-fold increase in ferritin and transferrin saturation were 1.04 (0.87-1.25) and 1.55 (0.98-2.45), respectively. After stratifying by cholesterol levels, the multivariable adjusted odds ratios (95% confidence intervals) for PAD associated with a two-fold increase in ferritin and transferrin saturation were 0.66 (0.41-1.05) and 0.75 (0.44-1.28), respectively, for women with total cholesterol <200 mg/dL and 1.20 (0.95-1.51) and 2.07 (1.01-4.22), respectively, for women with total cholesterol ≥ 200 mg/dL (p-value for interaction was 0.05 for ferritin and 0.02 for transferrin saturation).

## Discussion

In this representative sample of US adult men and postmenopausal women, ferritin and transferrin saturation were not statistically significantly associated with PAD. After stratifying the analysis by cholesterol levels, the observed associations were consistent with a synergistic interaction between ferritin and transferrin saturation with cholesterol levels among men and women. Our findings were consistent with a modest association of ferritin and transferrin saturation with PAD, particularly among those with high cholesterol levels, but we cannot exclude a chance association.

In 1981 Sullivan [21] introduced the hypothesis that elevated iron levels, below the levels found in hemochromatosis, may increase cardiovascular risk. This hypothesis was based on the observation that premenopausal women had lower rates of heart disease compared to men and this difference narrowed in concert with increasing iron levels as women went through menopause. The human body carefully maintains iron homeostasis, as both iron deficiency and iron overload are harmful [8]. Nearly all iron in the human body is bound to other molecules since free ferrous iron is highly reactive and may catalyze free-radical oxidative reactions. Free ferrous iron may be involved in the Fenton or Haber-Weiss reactions and catalyze the formation of hydroxyl radicals, which have been implicated in low-density lipoprotein (LDL) cholesterol oxidation. As a consequence, it has also been hypothesized that excess iron may be more harmful in those with elevated LDL cholesterol than those with lower LDL cholesterol [8].

While there is limited information in the literature on the association of iron with PAD, several studies have evaluated the association of iron with carotid artery atherosclerosis. In the Bruneck Study of 826 men and women, high ferritin levels were associated with greater progression of carotid artery atherosclerosis after 5 years [11]. In the Bruneck Study, the ferritin-carotid artery atherosclerosis relationship was significantly stronger at higher levels of LDL cholesterol. Conversely, a cross-sectional study of 206 Finnish men [13] and a case-control analysis of 730 men and women participating in the Atherosclerosis Risk in Communities (ARIC) study found no association between ferritin and carotid artery atherosclerosis [12]. In the ARIC study, there was no evidence to support a synergistic interaction between ferritin and cholesterol; in fact, the cholesterol stratified results indicated a lower odds ratio of carotid atherosclerosis among those with higher cholesterol levels.

The association of biomarkers of iron metabolism with coronary heart disease or stroke has been a source of substantial controversy [18-20,22-28]. A meta-analysis of 12 prospective studies found no evidence to support an association between biomarkers of iron metabolism and coronary heart disease [29]. Three prospective studies investigating the association of measures of iron metabolism and myocardial infarction have found evidence of synergistic interaction with cholesterol levels [18-20], but other similar studies have failed to find evidence of interaction [22,24]. Recently, a multicenter randomized controlled trial was conducted with 1,277 men and postmenopausal women randomized to either a control group or to receive phlebotomy at six-month intervals to reduce body iron stores; at the end of the six year follow-up, all-cause mortality and the secondary endpoint of all-cause mortality plus nonfatal myocardial infarction and stroke were nonsignificantly lower in the phlebotomy group [30].

The findings from the present study need to be considered in the context of its limitations. Ferritin and transferrin saturation are acute-phase reactants and may not accurately reflect body iron stores. To minimize this potential source of confounding we adjusted for CRP. NHANES 1999-2002 is a cross-sectional study susceptible to reverse causation and selection bias and, therefore, we cannot determine causality in any observed associations. There were relatively few cases of PAD, limiting our ability to detect modest associations and subjecting our findings to substantial random variability. Also, when investigating a possible interaction between ferritin and cholesterol levels, the use of LDL cholesterol would have been preferable to total cholesterol, but fasting LDL cholesterol was only available in 47% of our study participants. Despite these limitations, the present study maintains a number of strengths. NHANES data were collected using a rigorous study protocol with extensive quality control procedures and with technicians trained and certified in all data collection procedures. Additionally, the results are representative of the US noninstitutionalized civilian population.

## Conclusion

In a large nationally representative sample of US adults, we found a modest association of ferritin and transferrin saturation with PAD, particularly among those with high cholesterol levels, but we cannot exclude a chance association. After stratifying the analysis by cholesterol levels, the associations were also consistent with a synergistic interaction of cholesterol levels with ferritin and transferrin saturation. Prospective cohort studies with adequate power to investigate ferritin-cholesterol interaction are needed to fully characterize the relationship between biomarkers of iron status, cholesterol, and measures of atherosclerosis.

## Abbreviations

PAD: peripheral arterial disease; ABI: ankle-brachial blood pressure index; NHANES: National Health and Nutrition Examination Survey; BMI: body mass index; CRP: C-reactive protein; HDL: high-density lipoprotein; eGFR: estimate glomerular filtration rate; LDL: low-density lipoprotein; ARIC: Atherosclerosis Risk in Communities.

## Competing interests

The authors declare that they have no competing interests.

## Authors' contributions

AM participated in the analysis and interpretation of the data and drafted the manuscript. JMFR and PM participated in interpretation of the data and revised the manuscript. EG conceived the study, participated in the analysis and interpretation of the data, and revised the manuscript. All authors read and approved the final manuscript.

## Pre-publication history

The pre-publication history for this paper can be accessed here:


